# Does the Internet Use Improve the Mental Health of Chinese Older Adults?

**DOI:** 10.3389/fpubh.2021.673368

**Published:** 2021-07-16

**Authors:** Lin Xie, Hua-lei Yang, Xue-yu Lin, Shi-ming Ti, Yuan-yang Wu, Shuo Zhang, Si-qing Zhang, Wan-li Zhou

**Affiliations:** ^1^Department of Population and Labor Economics, University of Chinese Academy of Social Sciences, Beijing, China; ^2^School of Public Administration, Zhongnan University of Economics and Law, Wuhan, China

**Keywords:** internet use, older adults, mental health, depression, China

## Abstract

**Purpose:** The Internet has become an important part of daily life. However, older adults in China remain digital refugees amid the rapid development of digital information technology. This study attempts to scientifically answer how Internet use affects the subjective welfare of older adults.

**Method:** Using data from the 2014 and 2016 China Longitudinal Aging Social Survey (CLASS), a combination of ordinary least squares, ordered logit regression models, and propensity score matching (PSM) models were used to analyze the effects of Internet use on the mental health of Chinese older adults.

**Results:** Our findings suggest that Internet use affects the mental health of older adults and increases the incidence of their depressive symptoms. These findings are robust to changing the key indicators, research method, and sample. Further heterogeneity analysis reveals that the negative effects on mental health are more evident for specific groups of older adults, such as those who are women, younger and middle-aged, high-income, non-rural Hukou, less educated, and living with others.

**Conclusions:** Cultivating the ability of older adults to use the Internet and maintain a rational attitude while doing so can prevent its negative impact on their life satisfaction. Moreover, it can improve their attitudes toward using the technology and reduce their anxiety.

## Introduction

Since the 1990s, the Internet and other information technologies have rapidly developed in China. The number of Internet users in China reached 940 million in June 2020, and the proportion of users aged 60 and above is increasing from 6.7% in March 2020 to 10.3% now (1). Meanwhile, the aging population of China is growing rapidly. It is predicted that the share of the population over 60 years old in China will reach 34.9% by 2050, by which time China will enter a stage of deep aging (2). How to deal with this accelerating aging and the increasing number of the older population is not only the focus of discussions in academia and the government but also a topic of concern in all sectors of society (3).

In this context, mental health issues, such as loneliness and depression, are receiving increasing attention, but their relationship with Internet use is uncertain. Internet use has enhanced the mental health of older adults by expanding the scope of their social interactions, enriching their life experiences, and increasing the frequency of contact with family, friends, and other social network members (4). The openness, anonymity, virtualization, and equality potential of the Internet promote social participation in current affairs. Internet use also promotes social and community participation among older adults and improves their level of social adjustment. This in turn enhances their psychological well-being (3, 5–8), especially for those who are withdrawn and less socially engaged (9, 10). Online chatting can reduce loneliness and decrease the likelihood of depression in older adults. Using data from the Health and Retirement Survey (HRS) in the United States from 2002 to 2008, Cotten et al. (7) found that older adults were 33% less likely to be depressed when using the Internet. Khalaila and Vitman-Schorr (11) found that Internet use can improve their quality of life directly or indirectly by reducing loneliness, with the indirect effect influenced by ethnicity and the direct effect influenced by the amount of time older adults spend with their families. Using data from the 2008 U.S. Health and Retirement Study, Heo et al. (12) found that Internet use reduced loneliness in older adults by enhancing their social support as a mediating variable, which in turn enhanced their life satisfaction and psychological well-being, with the help of structural equation modeling. A recent study by Haase et al. (13) has suggested that older adults can mitigate the psychological effects of social isolation through virtual socialization during a new crown epidemic.

Studies of older adults with mobility impairments found that the Internet plays an important role in increasing interpersonal communication, maintaining family bonds, and expanding social networks (9, 14–16). However, overindulgence, undesirable Internet behaviors, and the spread of negative information and emotions may reduce the social participation of older adults, causing loneliness, and negatively affecting their mental health (17–21). The Internet time substitution hypothesis suggests that using the Internet reduces time and opportunities for offline social interactions, resulting in more self-isolation, which is detrimental to the expression of emotions and social relationships (22). In severe cases, excessive Internet use may even induce psychological disorders such as depression (22). This is in line with the technological stress theory that excessive use of the Internet can lead to health deterioration due to addiction (20, 21, 23, 24). Both Internet addiction or overuse and dependence on online social media increase the health risks of users (23). In addition, Internet use increases social comparison for relative income levels and social status, which plays a decisive role in the well-being of an individual (25–28). The spread of the Internet can increase access of older adults to information, constantly raising the upper limit of the material needs of people, making it easier to compare themselves online with people from any country and background. Social climbing behavior based on this can bring about a sense of psychological loss and relative deprivation (29–31). Further, the information depression theory argues that access of people to a large number of negative news reports *via* the Internet can reduce their enthusiasm for political participation and lead to a continued decline in social trust, which negatively affects their mental health (32).

From the above literature review, it can be seen that while scholars around the world have researched the relationship between Internet use and the mental health of older adults, this topic has not yet attracted widespread attention from scholars in China. Moreover, whether the above conclusions are applicable in the Chinese context requires further discussion. Previous studies have mostly focused on one dimension (e.g., rural or urban), one region, or small samples, limiting the robustness of the findings. In terms of research methods, most studies were conducted using ordinary least squares, Oprobit, and logit models, with less attention paid to endogenous issues. This means the results might be biased due to omitted variables and self-selection issues. For example, older adults with good health may be more willing to learn and use the Internet, which may affect the reliability of the findings.

This paper examines the following questions: What kind of impact does Internet use have on older adults? Can it help them improve their mental health and cope with depression? Considering the variability in the demographics of older adults, are there differences in the impact of Internet use on their mental health? The answers to the above questions can better clarify the relationship between Internet use and depression among older adults. Moreover, they can enhance the well-being of older adults and promote sustainable economic and social development, while ensuring the achievement of the Two Centenary Goals.

Our study examined the relationship between Internet use and mental health among older adults in China, based on data from the 2014 and 2016 China Longitudinal Aging Social Survey (CLASS). We used propensity score matching (PSM) to solve the above endogeneity problem while making the findings more internally valid by replacing the indicators, the study sample, and using difference-in-difference (DID) for robustness testing. On this basis, while considering the differences in older adult groups, a heterogeneity analysis was conducted by gender, age, Hukou, income level, education level, and the number of companions, to analyze the differences in the effects of Internet use among older adults.

This study makes the following contributions. First, it systematically compiled the research results on the impact of Internet use on the mental health of older adults, explored the relationship between Internet use and mental health among older adults in China based on CLASS data, and sought to confirm the applicability of existing studies to the Chinese sample. This makes the current study more externally valid. Second, in contrast to previous studies, we used the PSM model to examine the effects of Internet use on mental health and further ensured the internal validity of the findings by changing indicators and samples and using DID models. Third, based on existing research, this study compared differences in the mental health of different groups of older adults to further clarify the relationship between Internet use and depression among older adults in order to provide a realistic basis for better guidance on using the Internet to enhance the mental health of older adults. Finally, in response to the research findings and the actual situation, we make relevant policy recommendations, which may present an important reference for countries around the world in regulating Internet use.

The remainder of this paper proceeds as follows. Section Methods introduces data sources, variable selection, and the setting of the econometric model. Section Results focuses on the discussion of the research results, using the PSM method to study the relationship between Internet use and mental health among older adults and conducting a robustness analysis. Section Discussion shows a heterogeneity analysis to discuss the differences in the effects of Internet use on mental health among different groups of older adults. Section Conclusion concludes and presents the policy recommendations.

## Methods

### Data

The data used in the present study come from the CLASS, a nationally representative and longitudinal survey of Chinese aged 60 and above. CLASS is a nationwide, continuous, and large-scale social survey project whose objective is to regularly collect data on the social and economic background of the older adult population of China to understand the various problems and challenges they face in the process of aging, access the actual effects of various social policy measures in improving the quality of life of older adults and provide an important theoretical and factual basis for solving aging problems in China. This survey used a multistage sampling method. County-level units within provinces were selected as the primary sampling units (PSUs). A community or village was selected as the secondary sampling unit (SSU), and the final sampling unit was household. PSUs were randomly selected using a proportionate-to-population size sampling technique from a sampling frame containing all county-level units. The selection of SSUs followed the sample procedures as PSUs, and the ratio of urban to rural relevant population size was set at 6:4. People aged 60 years and above were randomly selected from each SSU based on a sampling map. The CLASS conducted its first nationwide survey in 2014 and two follow-up surveys in 2016 and 2018. Given that data from the 2018 wave have not yet been released, we only used data from the years 2014 and 2016.

The original sample size of the 2014 CLASS data was 11,511, with detailed information collected on key indicators of basic status, physical health, social participation, and social support of older adults. The final sample of 476 villages/neighborhoods in 134 counties corresponded to 28 out of 31 provinces (or municipalities) in China (CLASS webpage 1). After screening for variables and removing missing values, 7,040 respondents from 28 provinces presented sufficient data for the analysis. The 2016 CLASS data were based on a follow-up survey conducted on the 2014 data, successfully tracking 6,603 people, with a 57.4% follow-up rate, and after supplementing the sample with 4,892 people, the final sample size was 11,471 people. In addition, for the first time in 2016, CLASS included a survey on Internet use among older adults, which comprehensively measured key indicators affecting the lives of older adults. After variable screening and data cleaning, the final sample size of the regression model in this study was 6,972.

### Variables

#### Dependent Variable

Mental health was the dependent variable for this paper, referred to in CLASS as depressive tendencies. In the CLASS questionnaire, depression scores were calculated using the Depressive Tendency Scale (DTS), a simplified version of the CES-D scale, with nine questions covering aspects of daily mood, loneliness, sleep, sufficiency, and life status of older adults. Each question has three answers, “not,” “sometimes,” and “often,” with values of 1, 2, and 3, respectively. The scores of the nine questions are summed up and scored on a scale of 9–27, with higher scores indicating more severe depressive tendencies. Jin and Zhao (3) summed up the nine questions and used the higher score as a criterion for depressive tendencies; however, He et al. (33) found that the flow center depression scale (nine-question Chinese short version), with the reliability and validity of 17 points as the cutoff for distinguishing high risk of depression, was better. Therefore, in this study, we used two ranges, 9–17 and 18–27, and assigned the depressive tendency variable as 0 and 1, with 0 representing a low depressive tendency and 1 representing high depressive tendency. Based on a comprehensive evaluation of the multidimensional life of surveyed individuals, Ma (32) points out that life satisfaction is a stable measure of the long-term well-being of people. In the CLASS questionnaire, life satisfaction was divided into five levels, corresponding to values 1 (very satisfied) to 5 (very dissatisfied).

#### Independent Variable

The independent variable in this study was Internet use (*net)*. Referring to the study by Jin and Zhao (3), it was set as a dichotomous variable based on the CLASS question, “Do you often use the Internet now?” Moreover, given the prevalence of smartphone use today, smartphone use (*smart*) was regarded as a proxy variable of Internet use. The variable obtained from the question, “Do you currently use a smartphone?,” with a value of 1 for smartphone use and 0 for no smartphone use.

#### Covariates

Considering the influence of other factors on the mental health of older adults, gender, age, marriage, education, nation, religious belief (*religious*), Hukou, number of companions (*com_num*), income, whether they receive pension insurance (*pension*), level of community services (*com_s*), social support (*soc_s*), willingness to participate in society *(soc_p*), and number of children (*cld_num*) were used as covariates in the regression model. The results are shown in Table 1.

**Table 1:** Descriptive univariate information for variables.

**Variables**	**Descriptive univariate information**
**Dependent variables**	
Mental health	Low depressive tendency = 0, high depressive tendency = 1
Life satisfaction	Very satisfied=1, relatively satisfied=2, average=3, relatively dissatisfied=4, very dissatisfied=5
**Independent variables**	
Net	Internet use, using the Internet = 1, not using the Internet = 0
Smartphone	Smartphone use, yes = 1, no = 0
**Covariates**	
Gender	Male = 1, Female = 0
Age	Age of respondents
Marriage	Married with spouse = 1, other = 0
Education	Primary school and below = 0, junior school and above = 1
Nation	Han Chinese = 1, minority nation = 0
Religious	Religiously affiliated = 1, Not religiously affiliated = 0
Hukou	Rural = 1, non-rural = 0
*Com_num*	Number of people living permanently with the respondent
Health	Level of physical health, Very healthy = 1, relatively healthy = 2, average = 3, relatively unhealthy = 4, very unhealthy = 5
*Soc_p*	Willingness to participate in society; the higher the value, the stronger the willingness to participate in society
Income	Annual income of respondents
Pension	Receiving basic pension insurance = 1, not receiving basic pension insurance = 0
*Com_s*	Level of community services, the lower the value, the higher the level of community service
*Soc_s*	Social support, the higher the value, the higher the level of social support
*Cld_num*	Number of living children

### Model

The research question in this paper concerns the impact of Internet use on the mental health of older adults. However, Internet use by older adults is not random and may be subject to selective bias. If we simply use regression analysis, the estimates obtained may be biased. Therefore, we used the PSM method, which is an analytical method based on the counterfactual inference framework model proposed by Rosenbaum and Rubin in 1983 that can effectively address the problem of endogeneity. The basic idea is to compress the information collected from the multisample survey through logit regression or probit methods to produce a propensity score, and then match the treatment and control groups in the sample based on the propensity score to calculate the average treatment effect on the treated (hereafter referred to as ATT). In this study, ATT on the mental health of older adults was estimated by matching groups of older adults according to whether they use the Internet. In this study, the dummy variable *D*_*i*_ = {0, 1} is used to indicate whether older adults use the Internet, where 1 is the treatment group, representing older adults who use the Internet, and 0 is the control group, representing those who do not. The index of the extent to which mental health of older adults is affected by Internet use is expressed as *y*_*i*_. The treatment effect of *D*_*i*_ on *y*_*i*_ is:

(1) $$y_{i}=\{\begin{array}{c} y_{1i}\;D_{i}=1\\ y_{0i}\;D_{i}=0 \end{array}$$

where *y*_1*i*_ denotes the mental health of older adults who use the Internet, and *y*_0*i*_ denotes the mental health of those who do not. The treatment effect of Internet use on the mental health of older adults is

(2) $$\begin{array}{l} y_{i}=\left(1-D_{i}\right)y_{0i}+D_{i}y_{1i}=y_{0i}+\left(y_{1i}-y_{0i}\right)D_{i} \end{array}$$

The average treatment effect for participants is.

(3) $$\begin{array}{l} ATT=E\left[y_{1i}-y_{0i}|D_{i}=1,P\left(X\right)\right]=E\left[y_{1i}|D_{i}=1,P\left(X\right)\right]\\ \;-E\left[y_{0i}|D_{i}=1,P\left(X\right)\right] \end{array}$$

## Results

### Statistical Description of Variables

Table 2 shows the descriptive statistics of each variable, which are divided into three parts: the total sample, the treatment group (using the Internet), and the control group (not using the Internet). Their means and standard deviations were counted separately. As shown in Table 2, overall, only 11.3% of the total sample of older adults uses the Internet, while the relative proportion of older adults using smartphones is a little higher, reaching 17.5%. This shows that Internet use among older adults in China is still relatively low. In addition, the overall depressive tendency and life satisfaction of older adults in China were 0.273 and 2.157, respectively. Their mental health was good and life satisfaction was also at a relatively satisfactory level. To visually compare the differences in variables such as propensity to depression between Internet users and non-users, the statistics are presented separately in this paper. As shown in Table 2, the difference is 0.071 in depressive tendencies between Internet users and non-users, with Internet users having a higher depressive tendency index and poorer mental health. However, in terms of well-being in life, the sample group using the Internet was 0.285 lower than that of non-users and had a relatively lower sense of well-being. From the description of the variables, it can be intuitively seen that there are large differences between Internet users and non-users in terms of age, level of education, type of Hukou, income level, etc. Internet users are 3 years younger than non-users, but their level of education is 0.369 years higher and their income level is almost 16,000 RMB higher than non-users. These findings provide a basis for future research.

**Table 2:** Descriptive statistics of the main variables.

**Variables**	**Total sample** ** (*****N*** **=** **69,72)**		**Using the internet** ** (*****N*** **=** **791)**		**Not using the internet** ** (*****N*** **=** **6,181)**	
	***Mean***	***Sd***	***Mean***	***Sd***	***Mean***	***Sd***
Mental health	0.273	0.446	0.345	0.476	0.264	0.441
Life satisfaction	2.157	0.797	1.918	0.630	2.187	0.811
Net	0.113	0.317	1	0	0	0
Smartphone	0.175	0.380	0.891	0.311	0.0836	0.277
Gender	0.511	0.500	0.496	0.500	0.513	0.500
Age	70.26	7.544	67.04	6.786	70.67	7.538
Marriage	0.714	0.452	0.794	0.405	0.703	0.457
Education	0.338	0.473	0.665	0.472	0.296	0.457
Nation	0.752	0.432	0.824	0.381	0.742	0.437
Religious	0.0818	0.274	0.0973	0.297	0.0798	0.271
Hukou	0.442	0.497	0.119	0.324	0.484	0.500
*Com_num*	2.660	1.262	2.692	1.203	2.656	1.270
Health	2.638	0.937	2.255	0.860	2.687	0.936
Income	22405	59480	36629	31511	20584	61923
Pension	0.767	0.423	0.866	0.341	0.754	0.431
*Com_s*	17.87	0.664	17.77	0.876	17.89	0.630
*Soc_s*	14.31	5.549	14.03	5.619	14.34	5.540
*Soc_p*	22.58	7.420	25.28	5.060	22.23	7.601
*Cld_num*	2.492	1.391	1.777	1.117	2.584	1.396

### Baseline Regression Results

In this study, the treatment group (using the Internet) and the control group (not using the Internet) were used for PSM, and three matching methods were used: k-nearest neighbor matching, radius matching, and kernel matching. The results are shown in Table 3, with a mean treatment effect of 0.081 before matching; the results were significant at the 1% level. This indicates that, without controlling any variables, using the Internet increases the propensity of older adults to depression by 8.1%. In K-nearest neighbor matching, the depressive tendency was around 0.081 higher among older adults who used the Internet compared to those who did not, and the result was significant at the 1% level. To determine the accuracy of the results, the data were matched using both radius matching and kernel matching, and the average treatment effects obtained from matching were similar and significant at the 1% level.

**Table 3:** Propensity score matching (PSM) estimation for effect of Internet use on mental health.

**Matching method**	**Sample**	**Using the internet**	**Not using the internet**	**ATT**	**S.E**.
K-nearest neighbor (*n* = 4)	Before matching	0.345	0.264	0.081***	0.017
	After matching	0.345	0.364	0.081***	0.022
Radius matching	Before matching	0.345	0.264	0.081***	0.017
	After matching	0.345	0.270	0.075***	0.020
Kernel	Before matching	0.345	0.264	0.081***	0.017
	After matching	0.345	0.270	0.075***	0.020

*p < 0.1, **p < 0.05, ***p < 0.01; Standard errors after matching were obtained by the bootstrap method, and the number of self-help samples is 500.

To measure the balance between older adults who use the Internet and those who do not, that is, to observe whether there is a significant difference in the distribution of matching variables between the matching samples, we conducted a balance test on the PSM results. As shown in Table 4, the deviation proportion of all matching variables after matching decreased compared with that before matching, and the absolute error value of other variables decreased by more than 70%, except for some variables such as religious beliefs and community support. As the *t*-test results show, the hypothesis that the difference in matching variables between the two sample groups is zero cannot be rejected, which indicates that PSM greatly reduces the difference between the two samples of older adults, passing the balance test.

**Table 4:** Covariates balance testing for propensity score matching.

**Variables**	**Before matching**	**Mean value**		**Deviation%**	**Deviation reduction ratio%**	***T*****-test**	
	**After matching**	**Using the Internet**	**Not using the Internet**			***T*-value**	***P* > |*t*|**
Gender	U	0.496	0.513	−3.400	3.100	−0.900	0.369
	M	0.496	0.512	−3.300		−0.650	0.514
Age	U	67.04	70.67	−50.70	97.20	−12.90	0
	M	67.04	67.14	−1.400		−0.310	0.759
Marriage	U	0.794	0.703	21	84.60	5.310	0
	M	0.794	0.808	−3.200		−0.690	0.489
Education	U	0.665	0.296	79.40	98.60	21.31	0
	M	0.665	0.670	−1.100		−0.210	0.831
Nation	U	0.824	0.742	20	89.20	5.020	0
	M	0.824	0.833	−2.200		−0.470	0.641
Religious	U	0.0974	0.0798	6.200	−15	1.700	0.0890
	M	0.0974	0.0771	7.100		1.430	0.154
Hukou	U	0.119	0.484	−86.60	99.70	−20	0
	M	0.119	0.118	0.300		0.080	0.938
*Com_num*	U	2.692	2.656	2.900	43.50	0.750	0.453
	M	2.692	2.712	−1.600		−0.330	0.744
Health	U	2.255	2.687	−48	97.10	−12.31	0
	M	2.255	2.243	1.400		0.300	0.765
Income	U	36,629	20,584	32.70	92.30	7.170	0
	M	36,629	35,394	2.500		0.270	0.784
Pension	U	0.866	0.754	28.80	86.40	7.020	0
	M	0.866	0.851	3.900		0.870	0.387
*Com_s*	U	17.77	17.89	−14.70	75.10	−4.470	0
	M	17.77	17.80	−3.600		−0.600	0.546
*Soc_s*	U	14.03	14.34	−5.600	58.20	−1.490	0.137
	M	14.03	13.90	2.300		0.470	0.640
*Soc_p*	U	25.28	22.23	47.20	97	10.98	0
	M	25.28	25.38	−1.400		−0.350	0.729
*Cld_num*	U	1.778	2.584	−63.80	99.40	−15.62	0
	M	1.778	1.772	0.400		0.090	0.925

**Figure 1:**
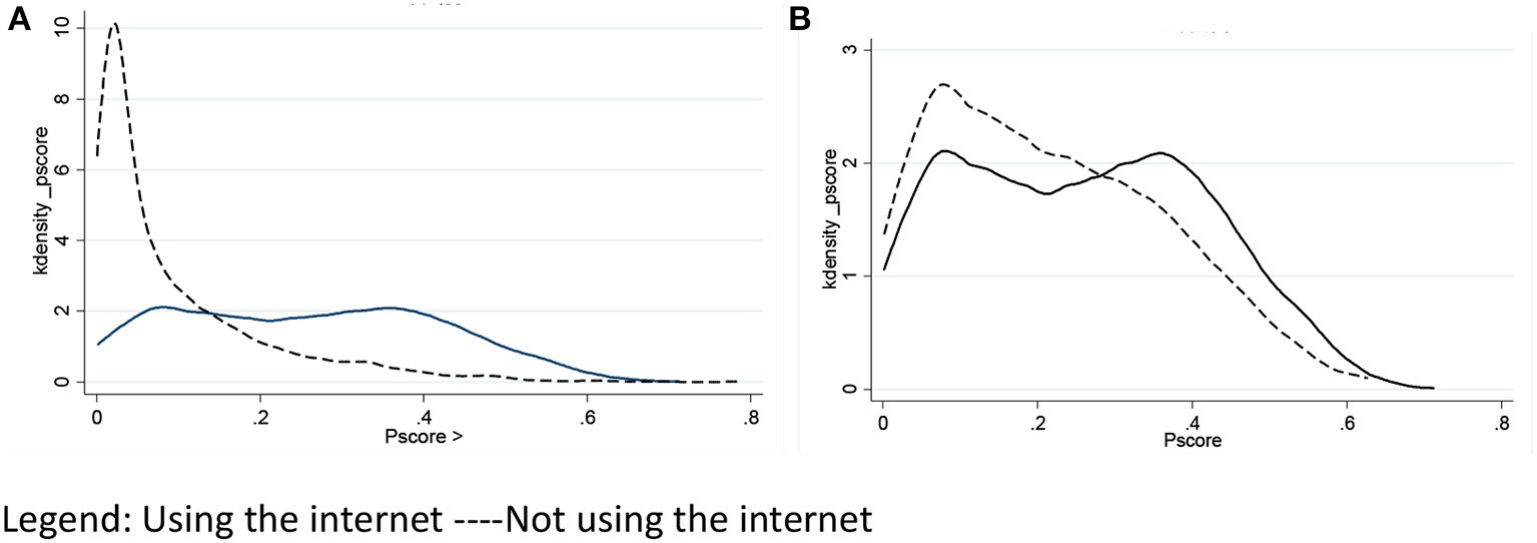
Kernel density maps: **(A)** before matching; **(B)** after matching. Using the internet—Not using the internet.

This study also reports the kernel density map before and after matching, as shown in Figure 1. After matching, the coincidence degree of the two curves in the treatment and control groups significantly improved.

In the CLASS questionnaire, a question on whether older adults use smartphones was included. Jin and Zhao (3) considered that the proportion of Internet users using mobile phones to access the Internet reached 99.1%. Therefore, the use of smartphones was regarded as an alternative variable of Internet use. Combined with the statistical data of this study, the number of older adults who use smartphones was 6% higher than those who use the Internet. Therefore, it was more representative to select smartphone use as an alternative variable to Internet use in this study for robustness tests. The regression results are shown in Table 5. Before matching, the average processing effect was 0.063, which was significant at the 1% level. Older adults using smartphones showed an increased depressive tendency and a worse mental health status. After matching, the average treatment effect was approximately 0.067, which was significant at the 1% level. Compared to before matching, the change in life satisfaction was small, but the results still showed that the use of smartphones has a negative impact on the mental health of older adults.

**Table 5:** Propensity score matching estimation for effect of smartphone use on mental health.

**Matching method**	**Sample**	**Using the internet**	**Not using the internet**	**ATT**	**S.E**.
K-nearest	Before matching	0.325	0.262	0.063***	0.014
neighbor (*n* = 4)	After matching	0.324	0.257	0.067***	0.018
Radius matching	Before matching	0.325	0.262	0.063***	0.014
	After matching	0.324	0.260	0.065***	0.017
Kernel	Before matching	0.325	0.262	0.063***	0.014
	After matching	0.324	0.260	0.065***	0.017

*p < 0.1, **p < 0.05, ***p < 0.01; Standard errors after matching were obtained by the bootstrap method, and the number of self-help samples is 500.

To reduce the impact of endogenous problems and avoid reverse causality as much as possible, we used the DID method to analyze the impact of Internet use on the mental health of older adults. Based on the CLASS data from 2014 to 2016 for analysis, the data of 2014 were treated as before Internet use and 2016 as after. It should be noted that in the DID model, we assigned a value of 0 for depressive tendencies and 1 for non-depressive tendencies. Table 6 presents the descriptive statistics of the variables.

**Table 6:** Descriptive statistics of variables using the DID method.

**Variables**	**Variable assignment**	**Mean** ** (*N* = 3,840)**	**S.E**.
Gender	Male = 1, female = 0	0.508	0.500
Nation	Han = 1, Minority = 0	0.936	0.244
Marriage	Married with spouse = 1, other = 0	0.659	0.474
Education	Primary = 0, Upper Secondary = 1	0.651	0.477
Religious	With religious beliefs = 1, no religious beliefs = 0	0.082	0.274
*Com_num*	Number of permanent residents with respondents	2.876	1.516
Hukou	Rural = 1, non-rural = 0	0.528	0.499
Work	Work status, work with income = 1, no job = 0	0.143	0.350
Pension	Getting basic endowment insurance = 1, not receiving basic endowment insurance = 0	0.580	0.494
*Com_s*	Level of community services; the lower the value, the higher the level of community service	17.83	0.956
*Soc_s*	Social support; the higher the value, the higher the degree of social support	13.59	5.979
*Cld_num*	Number of living children	2.746	1.483
Mental health	Depression tendency = 0, no depression tendency = 1	0.839	0.368
*Soc_p*	The higher the value, the stronger the willingness for social participation	19.58	10.68
Life satisfaction	Very satisfied = 1, relatively satisfied = 2, general = 3, less satisfied = 4, very dissatisfied = 5	2.131	0.902
Health	Very healthy = 1, relatively healthy = 2, general = 3, relatively unhealthy = 4, very unhealthy = 5	2.798	1.061
Net	Using Internet = 1, not using Internet = 0	0.095	0.293
Smartphone	Use smartphone = 1, not use smartphone = 0	0.157	0.364
Time	Year 2014 = 0, year 2016 = 1	0.500	0.500
Treated	Using Internet = 1, not using Internet = 0	0.095	0.293
gd	gd = time*treated	0.047	0.213

The results of the DID method are shown in Table 7. Before using the Internet, the difference between the experimental and control groups was 0.016. After using the Internet, the difference between the experimental and control groups was 0.082. The final DID result was −0.099, which was significant at the 5% level. This means that the average depression tendency of Internet users increased by 0.099 and their mental health status decreased, which is consistent with previous results.

**Table 7:** Difference-in-difference (DID) estimation for the effect of Internet use on mental health.

	**Depression tendency**	**Standard error**	**|*t*|**	***P* > |*t*|**
**Before using the internet**				
Control group	1.026			
Treatment group	1.042			
Diff (T–C)	0.016	0.016	1.040	0.299
**After using the internet**				
Control group	0.807			
Treatment group	0.725			
Diff (T–C)	−0.082	0.038	2.170	0.030**
Diff-in-Diff	−0.099	0.040	2.450	0.014**

*p < 0.1, **p < 0.05, ***p < 0.01.

To further verify the robustness of the results, we used the 2017 Chinese General Social Survey (CGSS) data to re-estimate. There were 12,582 original samples from the data. After deleting missing values, the sample size used in this study was 4,225. We also employed the PSM method. The question “How often have you felt depressed or were depressed in the past 4 weeks” replaced the depression tendency variable in this paper as the independent variable. The answers to the question were “always,” “often,” “sometimes,” “rarely,” and “never,” with values of 1 to 5, respectively. The higher the value, the better the mental state and lower the depressive tendency. According to the CGSS 2017 questionnaire, the question “Have you ever been online in the past six months, including using computers, mobile phones, smart wear, and other devices?” determined the dependent variable. In this study, it was set as a binary variable, with the same control variables, and the same method was used to deal with variables. Finally, 12 other control variables were retained: gender, marital status, education level, nationality, religious belief, Hukou type, number of accompanying persons, working status, whether receiving pension insurance, social participation and willingness, and number of children.

As shown in Table 8, before sample matching, the average treatment effect was 0.414, which was significant at the 1% level. After sample matching, the average treatment effect was about −0.03 to −0.07. This shows that Internet use can increase depression or the frequency of depression in older adults and has a negative impact on their mental health, which is consistent with the results obtained above.

**Table 8:** Results of the PSM and Chinese general social survey (CGSS) data.

**Method**	**Sample**	**Using the internet**	**Not using the internet**	**ATT**	**S.E**.
K-nearest	Before matching	4.057	3.643	0.414***	0.038
neighbor (*n* = 4)	After matching	4.057	4.131	−0.074	−1.03
Radius matching	Before matching	4.057	3.643	0.414***	0.038
	After matching	4.057	4.091	−0.034	0.069
Kernel	Before matching	4.057	3.643	0.414***	0.038
	After matching	4.057	4.093	−0.036	0.069

*p < 0.1, **p < 0.05, ***p < 0.01.

### Heterogeneity Analysis

From the results presented above, we can draw the conclusion that Internet use reduces mental health and increases depression in older adults. However, the above results are only the average effect of the whole sample analysis, and the differences among different groups of older adults are not considered. To further study the impact of Internet use on mental health among different groups of older adults, this study also analyzed heterogeneity by gender, age, income level, Hukou type, education level, and number of accompanying persons. Similarly, to avoid endogeneity problems, the analysis results are based on PSM. Three methods such as k-nearest neighbor matching, radius matching, and kernel matching were used. The results are shown in Table 9.

**Table 9:** Results of heterogeneity analysis (PSM estimation).

**Matching method**	**Gender**		**Age**			**Income levels**	
	**Male**	**Female**	**Low** ** (60–69)**	**Middle** ** (70–79)**	**High** ** (80 and above)**	**Less than RMB 22,405**	**More than RMB 22,405**
K-nearest neighbor (*n* = 4)	0.054^*^ (0.030)	0.088^***^ (0.033)	0.081^***^ (0.022)	0.081^***^ (0.022)	0.000 (0.076)	−0.010 (0.038)	0.127^***^ (0.027)
Radius matching	0.054^*^ (0.027)	0.096^***^ (0.030)	0.075^***^ (0.020)	0.075^***^ (0.020)	0.009 (0.070)	−0.003 (0.034)	0.116^***^ (0.025)
Kernel	0.054^**^ (0.027)	0.097^***^ (0.030)	0.075^***^ (0.020)	0.075^***^ (0.020)	0.014 (0.070)	−0.001 (0.034)	0.116^***^ (0.025)
	**Hukou**		**Education**			**Number of companions**	
	**Rural**	**Non-Rural**	**Primary**	**Upper secondary**		**Living alone**	**Not living alone**
K-nearest neighbor (*n*=4)	−0.066 (0.049)	0.097^***^ (0.024)	0.087^***^ (0.029)	0.029 (0.033)		0.072 (0.076)	0.084^***^ (0.023)
Radius matching	−0.060 (0.043)	0.092^***^ (0.022)	0.100^***^ (0.026)	0.030 (0.030)		0.077 (0.070)	0.084^***^ (0.021)
Kernel	−0.060 (0.043)	0.093^***^ (0.022)	0.100^***^ (0.027)	0.029 (0.029)		0.074 (0.070)	0.085^***^ (0.021)

*Standard errors are reported in the parentheses*;

* *p < 0.1*,

** *p < 0.05*,

*** *p < 0.01*.

In the heterogeneity analysis by gender, the average treatment effect for men was 0.054, significant at the 10% level, while the average treatment effect for women was 0.088, significant at the 1% level.

In terms of Hukou differences, we analyzed the overall older adult population as rural and non-rural samples for empirical purposes. Results show that the depressive tendencies of older adults with rural Hukou decreased by ~6%, though it should be noted that the results are not significant. Contrastingly, the depressive tendencies of older adults in the non-rural group increased by about 9%, and the average processing effect was significant at the 1% level; therefore, it can be stated that the impact of Internet use on the mental state of older adults with non-rural Hukou is more pronounced.

In terms of differences in educational level, we divided the sample into two groups: primary and upper secondary education. The results showed that the average processing effect of the primary education sample was ~10%, significant at the 1% level and that for the intermediate and higher education sample was 3%.

In terms of the number of companions, we divided them into older adults living alone and those not living alone. The results showed that the average processing effect of Internet use for the older adults living alone was 7%, while that for the non-older adult group was 8%, significant at the 1% level.

## Discussion

The Internet has a negative impact on the mental health of older adults, increasing their depressive tendencies. Nie and Erbring (10) argued that the Internet reduces opportunities for face-to-face communication with other members of society, leading to a sense of isolation, which is detrimental to the expression of emotions and the maintenance of social relationships. Using experimental data from 169 participants in 73 households, Kraut et al. (25) found that as Internet use increases, communication with family members decreases, while levels of loneliness and depression increase. This indicates that Internet use may replace some real-life social activities, which reduces social participation and produces negative psychosocial effects. Moreover, as the social circles of older adults continue to shrink due to factors like declining physical ability, Internet use can significantly increase their levels of loneliness.

Due to limited socialization after retirement in China, changes in the family structure with children leaving home for work, and fewer opportunities for social activities, face-to-face communication has been greatly reduced in the lives of older adults. Therefore, Internet use might further lessen their opportunities for emotional expression. The social support theory suggests that older adults receive material and emotional support by communicating and interacting with people or groups within their own social network, which enhances their sense of well-being. The mental health of older adults can be seriously affected when they lack emotional support from Internet use, resulting in negative emotions, such as depression. At the same time, when loneliness cannot be effectively alleviated in real life, older adults are likely to become overdependent on the Internet, resulting in addiction. The sense of emptiness and loss they feel when they return to reality after too much time on the Internet can further endanger their mental health.

Due to the late popularization of the Internet, the elderly in China often have poor computer literacy, which refers to the ability to use computers and software to complete practical tasks. Poor computer literacy can also affect their mental health in the process of use and difficulties in the process of operation can stimulate their anxiety. For example, while the elderly could get more information online to improve their mental health, negative content can mislead them, damaging their mental health. Yin and Neyens (34) found that about 62.3% of those with inflammatory bowel diseases reported they had looked up health information online, 16.3% reported they had scheduled an appointment with a health care provider online, and 21.6% reported having used a computer to communicate with a health provider by email.

Internet use is more likely to cause depression and has a stronger negative effect on the mental health of older women than that of men. This is consistent with the findings of Yang and Lester (35), who reported that it was because older women were less skillful, and Schumacher and Morahan-Martin (36) found the older women had not received a good education and were different from men in terms of computer operation and its related aspects. In terms of age, this study was based on the treatment applied in the study by Peng et al. (37), which divided the population of older adults into three age groups: low (60–69 years old), middle (70–79 years old), and high (80 years old and above). The results are shown in Table 9, with the average treatment effect for “low” and middle-aged older adults being similar at around 0.08, both significant at the 1% level, while that for older adults was close to 0. Compared with older adults, the physical condition of middle-aged and young people is more ideal as they have more energy and find it easier to learn Internet skills; thus, the impact of Internet use is more evident. However, as older adults enter the advanced-age stage, their physical health worsens and they use the Internet less frequently. In the statistical samples, older adults only account for 0.3% of the sample data on Internet use, which is 2.6% of the total number of older adults who use the Internet. Therefore, the effect of Internet use was not evident.

This study used income level as a variable to investigate the impact of Internet use on the propensity score for depression in older age groups with different incomes because it is an important factor influencing the subjective well-being of an individual (38). The income groups were classified into two—below-average and above-average—based on the criterion that the average annual income is approximately RMB 21,792 (39). Table 9 shows that the average treatment effect for the high-income group was about 0.1, higher than the low-income group, and is significant at the 1% level. This may be because financially stable older adults tend to use the Internet more (40) and thus suffer more pronounced shock effects. Moreover, people have the ability to adjust to changes in their environment. When their income increases, their expectations also rise and they quickly and automatically adapt to their increased income (41, 42). Therefore, as expectations rise and the desire for material well-being increases, Internet use allows people to more easily compare the lives of individuals with their own, creating a comparison effect and reducing their sense of well-being (28). Wu (43) argued that older adults have access to a more comprehensive range of information through the Internet. The influence of such information has led to more social comparisons among older adults, causing a decline in life satisfaction.

The difference between urban and rural may be due to the long-term influence of the urban–rural dualistic structure. In rural areas, Internet penetration is lower and the educational level of older adults is also relatively lower than their urban counterparts, which leads to a lower impact on their mental health. Ma and Le (44) indicated that these differences were caused by the late development of the Internet in rural areas, coupled with traditional concepts of production and life of premodern rural residents. Another reason is that urban older adults are more likely to be exposed to a more exciting world due to higher Internet usage. They are also more easily influenced by their new experiences, such as finding out about the lives of others through social networks and friend circles. However, discovering that others are better off than them can cause feelings of disappointment and loss. Due to the lack of a fixed retirement age among rural older adults, they still engage in agricultural labor after the age of 60, as opposed to those with non-rural Hukou. This enriches their activities in their later lives, leaving them with fewer inner desires. It also provides them with better mental health and life satisfaction.

Internet use has a greater impact on older adults with lower levels of education, increasing the likelihood of depression. This is in line with the findings of Peng et al. (37) who found that the effect of Internet use on subjective well-being is inhibited in older adults by primary school qualifications. It is possible that the effect is more pronounced because older adults with lower levels of education are less able and more reluctant to learn new technologies; they also find the learning process to be more complicated. In addition, anonymity on the Internet has become a “severe disaster area” of junk information such as violence, pornography, gambling, cults, and so on. Using the Internet to carry out illegal and criminal activities, like network fraud, has become increasingly rampant (45). Cao (46) found that the ability of individuals with higher education levels to identify and obtain information is greater than that of those who are less educated. The former has a knowledge advantage that enables them to search for useful information or distinguish between true and false information. Thus, older adults with lower education levels are more vulnerable to the negative effects of the Internet.

Internet use can disrupt the real-life relationships of older adults who do not live alone and spend more time with online interactions, than cultivating strong in-person ties (38). However, the negative impact of excessive Internet use on the mental health of older adults living alone was not evident due to their lack of companionship.

## Conclusions

In light of the rapid spread of the Internet and mental health of older adults in China, we examined whether the use of the Internet can improve their mental health to help China achieve the goal of active aging and the Two Centenary Goals. We used the 2014 and 2016 CLASS data to scientifically answer this question. Our analysis yielded the following results. Overall, Internet use has a negative impact on the mental health of older adults, specifically an increased tendency to develop depression. To reduce the effect of endogenous problems, this conclusion still holds after a robustness analysis with the addition of a sample, a change of methodology, and a change in the sample. Considering the possible differences between different groups of older adults, we analyzed heterogeneity by age, gender, Hukou, income level, education level, and number of companions and found that Internet use has a stronger negative effect on mental health and is more likely to lead to depression in female older adults in the middle and lower age groups, high-income group, non-agricultural group, less educated group, and the group of older adults who do not live alone.

Based on these empirical results, we provide certain recommendations and insights in the following six areas to promote the rational use of the Internet by the older adult population of China in order to ensure a happy and active old age.

Improving attitudes toward Internet use among older adults and reducing their fear of the Internet. Raising awareness and improving attitudes toward Internet use can motivate older adults to use the Internet and reduce their fears. For example, Internet use can be integrated into community activities for older adults to increase their knowledge of the Internet and thus improve their attitudes toward its use. Older adults should adapt their mindset and take the initiative to learn the skills to use the Internet and related smart products to overcome their anxiety.

Developing the ability of older adults to use the Internet and reducing the sense of powerlessness in Internet use. In the age of the Internet, older adults are called digital refugees due to the limitations resulting from their educational background, behavioral habits, and age. They are generally less able to use the Internet and may even have a sense of technological panic, which seriously affects their physical and mental health. A targeted training service to building the capacity of older adults to use the Internet will help them bridge the digital divide and achieve active aging. Moreover, mobilizing the strength of society and family members through educational feedback and peer learning can effectively enhance the ability of older adults to use information tools.

Enhancing experience of older adults in using the Internet and, thus, their sense of well-being: Target audience of modern technology is mainly young people; however, there are significant differences between older adults and younger people in terms of physical function and psychological awareness, and their product designs are not suitable for older adults. For example, cluttered page layouts, small web fonts, and inappropriate content give older adults a poor user experience and damages their physical and mental health. Therefore, existing devices and applications need to gradually incorporate age-friendly design, especially considering the declining visual and auditory abilities of older adult users, and make age-appropriate changes in voice, text recognition, font size, etc., to enhance the experience of older adults.

Regulating the use of Internet content by older adults. Older adults, as a special group, are susceptible to the influence of inappropriate content on the Internet, which can be detrimental to their supervision of relevant content accessed by older adults when using the Internet and make use of big data to better tap the potential needs of older adults and provide them with targeted services and products that meet their actual needs.

Reasonable guidance for older adults using the Internet. Inappropriate use of the Internet refers to excessive use due to an inability to control the online behavior of an individual, which leads to significant psychological depression and waste of time, as well as failures in social interactions and family relationships. Internet addicts have less time for face-to-face interactions, which seriously affects their daily lives, interpersonal relationships, and sense of psychological well-being (47). Therefore, older adults, especially those with poor self-control, need to be reasonably guided in Internet use in order to develop good Internet habits, ensure a healthy lifestyle, take advantage of the positive effects of the Internet, and reduce its negative impact on their health.

Developing different support policies for different age groups. Through the heterogeneity analysis above, different groups of older adults should be provided with targeted services; for example, rural and less educated older adults, especially women, could be better trained to use the Internet and older adults living alone given priority to help develop relevant skills to meet their needs for social interaction, leisure, and entertainment.

The study has some limitations mainly related to the survey data. First, our analysis is limited to the sample, and caution is still needed when extrapolating our analysis to current situations. For that lifestyles of people, including their Internet use, have changed tremendously due to the pandemic contingency. Second, the measures used are relatively simple and do not allow for a more microscopic cognitive exploration of the psychological mechanisms at play between Internet use and mental health among older adults. In addition, the mechanisms and extent of the mental health effects are not yet clear. In spite of these, these limitations open up new research directions subsequent studies can consider and expand on. Further, this study provides a systematic analysis of the effect of Internet use on the mental status of older Chinese adults. It has important implications for Internet use promotion in China and other developing countries.

## Data Availability Statement

The raw data supporting the conclusions of this article will be made available by the authors, without undue reservation.

## Author Contributions

LX and H-lY conceived this research. S-mT was responsible for the methodology. LX conducted software analyses. SZ and S-qZ conducted necessary validations. Y-yW conducted a formal analysis and managed the investigation. X-yL and S-mT gathered resources, curated all data, wrote/prepared the original draft, and were responsible for project administration. LX and W-lZ reviewed and edited the manuscript, were responsible for visualization, supervised the project, and acquired funding. All authors contributed to the article and approved the submitted version.

## Conflict of Interest

The authors declare that the research was conducted in the absence of any commercial or financial relationships that could be construed as a potential conflict of interest.
